# Is Transcranial Alternating Current Stimulation Effective in Modulating Brain Oscillations?

**DOI:** 10.1371/journal.pone.0056589

**Published:** 2013-02-14

**Authors:** Debora Brignani, Manuela Ruzzoli, Piercarlo Mauri, Carlo Miniussi

**Affiliations:** 1 Cognitive Neuroscience Section, IRCCS Centro San Giovanni di Dio Fatebenefratelli, Brescia, Italy; 2 Department de Tecnologies de la Informació i les Comunicacions, Universitat Pompeu Fabra, Barcelona, Spain; 3 Department of Clinical and Experimental Sciences, National Institute of Neuroscience, University of Brescia, Brescia, Italy; University of British Columbia, Canada

## Abstract

Transcranial alternating current stimulation (tACS) is a promising tool for modulating brain oscillations, as well as a possible therapeutic intervention. However, the lack of conclusive evidence on whether tACS is able to effectively affect cortical activity continues to limit its application. The present study aims to address this issue by exploiting the well-known inhibitory alpha rhythm in the posterior parietal cortex during visual perception and attention orientation. Four groups of healthy volunteers were tested with a Gabor patch detection and discrimination task. All participants were tested at the baseline and selective frequencies of tACS, including Sham, 6 Hz, 10 Hz, and 25 Hz. Stimulation at 6 Hz and 10 Hz over the occipito-parietal area impaired performance in the detection task compared to the baseline. The lack of a retinotopically organised effect and marginal frequency-specificity modulation in the detection task force us to be cautious about the effectiveness of tACS in modulating brain oscillations. Therefore, the present study does not provide significant evidence for tACS reliably inducing direct modulations of brain oscillations that can influence performance in a visual task.

## Introduction

In the last decade, there has been a gathering consensus on the functional role of brain oscillations in a variety of cognitive functions and their importance for understanding brain processing [1], [2]. Evidence has been reported that most cognitive processes rely on the synchronous activity of large ensembles of functionally linked firing neurons, which occur in distinct frequency bands according to the extension of the network architecture [3], [4], [5], [6]. Recent years have seen the emergence of the exciting possibility of inducing the controlled entrainment of brain rhythms through the use of non-invasive brain stimulation techniques, such as repetitive transcranial magnetic stimulation (rTMS) and transcranial alternating current stimulation (tACS) [7], [8], [9]. In comparison to the classical rhythmic sensory stimulation protocols (e.g., visual flicker), the use of these methodologies offers the advantage of directly stimulating cortical targets and bypassing primary sensory structures and areas of the input pathways (sub-cortically and cortically). The possibility of externally manipulating brain oscillations allows inferences about the causal role of brain frequencies in cognition and also holds promise for therapeutic purposes. Indeed, abnormalities in neuronal synchronisation have been reported in many brain disorders, such as schizophrenia, epilepsy, autism, Alzheimer's and Parkinson's diseases [10], [11]. The opportunity to exogenously modulate these mechanisms could pave the way for new rehabilitative applications [9].

Strong evidence of the ability of rTMS to induce frequency entrainment has been recently reported. Thut and colleagues [12] showed that rTMS over the posterior parietal cortex causes a local entrainment of the preferred frequency of the underlying generator (i.e., alpha), as revealed by a combination of TMS–EEG recordings. Furthermore, other rTMS studies (designed on *a priori* EEG knowledge of strict relationships between particular frequency bands and cognitive functions) have showed topographic- and frequency-specific effects of rTMS on the behavioural performances of participants [13], [14], [15], [16].

Even if the method remains controversial, the possibility of directly entraining oscillations in the brain has also been suggested for tACS [17], [18], [19], [20], [21], [22]. Recently rediscovered in the survey of cognitive neuroscience, tACS involves applying weak alternating electrical currents to the head via two electrodes, which are usually both located on the scalp. Kanai and colleagues [17], delivered tACS over the occipital cortex, which induced visual experiences (i.e., phosphenes) that were ascribed to the direct interaction between tACS and the on-going oscillations of the primary visual cortex. In particular, beta frequency stimulation was more effective in inducing phosphenes in an illuminated room, whereas alpha frequency stimulation was more effective in darkness. Researchers have argued, however, that tACS-induced phosphenes may actually be the result of activation of the retina [23] instead of the primary visual cortex. Indeed, electrical current could reach the orbital area and retina via volume conduction of the scalp irrespective of electrode configurations on the head [24]. Evidence consistent with this interpretation was already reported in the last century, when Brindley [25] observed that the intensity needed to elicit phosphenes increased with the distance from the eye and that they cannot be elicited after pressure blinding. What deserves attention, however, is not merely concern about the retinal versus cortical origin of tACS-induced phosphenes, but the possibility of tACS directly stimulating the cortex (but see [26]). The controversy is not new. Lippold and Redfearn [27] observed that, to obtain “psychological effects at imperceptible current strengths”, the current flow needed to enter the orbital fissure. In addition, Smitt and Wegener [28] and Hayes [29] recorded intracerebral voltage measurements in human cadavers and alive monkeys, respectively, and found evidence for a general diffusion of current through the brain. Widespread changes in neuronal activities of cortical and subcortical regions after electrical stimulation have recently been reported with modern neuroimaging techniques [30], [31]. Therefore, if tACS is effective for cortical stimulation, its effects appear to be generalised to all of the brain, instead of localised to a single cortical area.

Even if controversial, investigating the ability of tACS to induce direct cortical modulations of the natural brain oscillations remains an important issue because transcranial electric stimulation (tES) offers some advantages over TMS in terms of safety. Additionally, tES has a better translation in the clinical setting because the method is more feasible [32], [33]. To this end, the current study was designed to exploit the well-established relation between posterior alpha-oscillations (8–14 Hz) and visual attention/perception [15], [34], [35], [36], [37]. A role in regulating the incoming information at early stages of processing has been assigned to the posterior alpha rhythm through the functional inhibition of task-irrelevant regions [38], [39], [40]. Specifically, posterior alpha power before stimulus presentation is inversely related to the quality of perception [15], [41], [42], [43], [44] and retinotopically organised in accordance with the focus of attention [34], [45], [46].

Based on this evidence, we decided to apply tACS at the alpha frequency (10 Hz) over the right or left occipito-parietal areas while participants performed a visual detection and discrimination task with targets appearing in one of the two visual fields with the same probability. A low (6 Hz) and a high (25 Hz) tACS frequency were used as control conditions (in addition to sham stimulation) to rule out possible unspecific effects of current stimulation. The hypothesis was straightforward. If tACS was able to induce a local entrainment of the alpha rhythm, the entrainment would mimic the natural brain rhythms of inhibition, leading to a decline of target perception in the visual field contralateral to the alpha-stimulated hemisphere. This rationale was the same as used by Romei and colleagues [15] in testing the possibility of entraining specific local frequencies with rTMS.

## Materials and Methods

### Participants

All participants were right handed according to the Edinburgh handedness inventory test [47], had normal or corrected-to-normal visual acuities and showed no risk factors for tACS application, as assessed through safety questionnaires. Each participant recruited for the study completed a preliminary behavioural session. Those who demonstrated ceiling (accuracy at ceiling of the second contrast) or floor effects (flat accuracy function through contrasts) were excluded. As a result, 96 healthy volunteers participated in the entire experiment. They were randomly assigned to one of four groups of stimulation, each one composed of 24 participants: sham (12 females; mean age = 22 years; SD = 2.3), 6 Hz (12 females; mean age = 21 years; SD = 2.4), 10 Hz (12 females; mean age = 22 years; SD = 3.2) and 25 Hz (12 females; mean age = 22 years; SD = 2.1). In each group, half of the participants received tACS over the right hemisphere and the other half over the left hemisphere. All the participants were naïve to tACS effects and did not know to which stimulation group they were assigned. The experimental method had the approval of the Ethics Committee of the IRCCS Centro San Giovanni di Dio Fatebenefratelli, Brescia, Italy. Written informed consent was obtained from each participant.

### Stimuli

Target stimuli were low-contrast Gabor patches (sinusoidal gratings of 0.94 cpd enveloped by a Gaussian) tilted 22 degrees to the left or right with a diameter of 3.39 degrees of visual angle. The stimuli had five different contrast levels, ranging from 0.034 to 0.052 (Michelson contrast). In a pilot experiment, we tested an additional group of twelve participants with a similar task, using the method of constant stimuli with seven contrast levels. In the main experiment, the central contrast was adjusted at the threshold level estimated in the pilot. Thus, two of the remaining contrasts were sub-threshold, and the other two were supra-threshold. We also implemented catch trials at 0.0 (Michelson contrast) to estimate the false alarm rate.

Stimuli were displayed on a Hanns.G LED monitor with a screen resolution of 1920×1080 pixels. The presentation was controlled by the Psychtoolbox package from Matlab (MathWorks, Natick, MA) [48], [49]. The mean luminance of the display was 55.9 cd/m^2^. Gamma correction was applied. The screen was at a distance of 56 cm from the participants.

### Procedure

**Figure 1:**
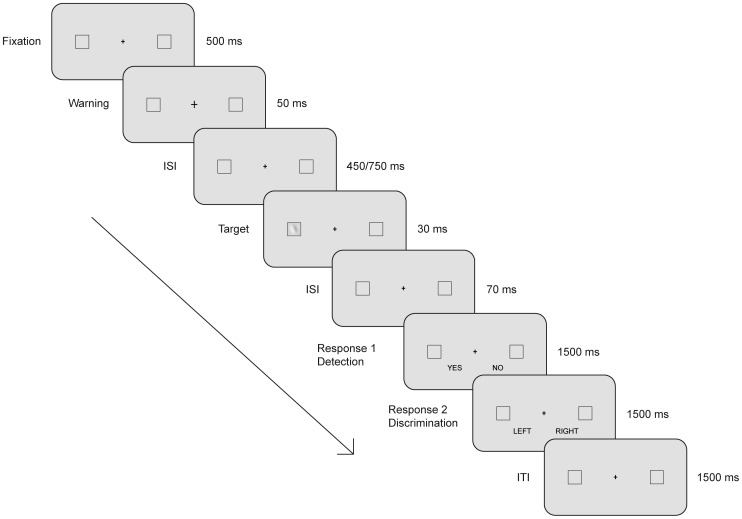
Schematic illustration of the temporal structure of a trial. A Gabor patch at different contrast levels appeared for 30 ms inside one of two lateral placeholders after a variable interval (450–750 ms) from a warning signal (the fixation cross became larger for 50 ms). Participants had to provide two consecutive responses: first, to report whether they believed the Gabor patch was present or not (detection) and then to select its orientation (discrimination). For every response, a fixed interval of 1500 ms was available.

The task is schematically displayed in figure 1. Participants were instructed to maintain fixation on a central cross. After a variable interval (450–750 ms) following a warning signal (i.e., the fixation cross became larger for 50 ms), a target appeared for 30 ms inside one of two placeholders (squares of 3.39 degrees) positioned at ±6 degrees eccentricity of the visual angle along the horizontal meridian relative to the central fixation cross (0.5 degrees). Two consecutive responses were required. The first response (R1-detection) was a yes/no stimulus detection task, in which the participants were asked to report whether they believed stimuli were present. The words “yes” and “no” were presented horizontally, 4.5 degrees below the central fixation cross, and participants were instructed to press one of the two corresponding response buttons on the keyboard (i.e., C or N) with their right or left index fingers. The respective positions of yes – no responses were balanced across participants. In the second response (R2-discrimination), participants were forced to decide between two orientation alternatives (±22 degrees). They were specifically asked to perform the orientation discrimination task in all trials, even when they did not believe a stimulus was previously present. All participants pressed the right response button (N) with their right index finger for clockwise orientation (+22 degrees) and the left response button (C) with their left index finger for counterclockwise orientation (−22 degrees). For every response, a fixed time limit of 1500 ms was given. However, accuracy was emphasised over speed. After an intertrial interval of 1500 ms, the next trial began.

All participants performed short training sessions to familiarise themselves with the task (16 trials) and then a baseline session, followed by a tACS session. In the baseline session, participants simply performed the behavioural task, while in the tACS session, they performed the same task concurrently with tACS application. Even if a learning effect occurred across sessions, a fixed order of the sessions was maintained to avoid any possible tACS carry-over effects in the blocks without stimulation. Both sessions consisted of 144 trials, including 12 targets in the left and right visual fields for each of the 5 contrast levels and 24 catch trials. Every session was divided into three blocks; each block had a mean duration of approximately 5 minutes interleaved with short breaks. Trials at different contrast levels within each block were presented in a randomised order. The entire experiment lasted approximately 75 minutes including the montage of tACS electrodes and breaks.

### Transcranial electrical stimulation - tACS

tACS was continuously delivered for the entire duration (5 min) of each block of the tACS session by a battery-driven current stimulator (neuroConn GmbH, Ilmenau, Germany) through conductive-rubber electrodes placed in sponges. A small target electrode (16 cm^2^) was placed (according to the participant group) over the left or right occipito-parietal areas (PO7 or PO8), as determined by the International 10–20 EEG system. The reference electrode was positioned over the vertex CZ and was larger than the occipito-parietal electrode (35 cm^2^) to reduce current density and limit stimulation effects under its surface [50]. The electrode's position was based on the montages in previous studies of the visual cortex [17], [51] and studies reporting tES effects in the hemifield contralateral to the stimulation side [52], [53]. A sinusoidal electrical current waveform with no DC offset was delivered at a particular frequency to each group (i.e., 6 Hz, 10 Hz or 25 Hz). The intensity was 1 mA (peak-to-peak) to avoid the perception of flickering lights usually reported with higher stimulation intensities [17], [20]. In the sham stimulation group, a 10 Hz tACS was turned off 10 s after the beginning. All the stimulation parameters (max current density = 0.063 mA/cm^2^; duration = 5 minutes×3 blocks; max total charge = 0.056 C/cm^2^) were maintained below the safety limits [54]. At the end of the experiment, all participants completed questionnaires [55] to evaluate possible discomforts induced by tACS and influences on their performances.

### Data Analysis

The behavioural measure of interest was accuracy (i.e., proportion of correct responses) as a result of the instructions emphasising perceptual aspects of the task. Arcsine-transformed accuracy [56] was determined separately for the first (R1) and second (R2) responses for each contrast level and stimulation group. Specifically, the computation of the R2 Accuracy (discrimination task) was independent of whether participants had or had not observed the Gabor patch in the first instance.

To verify whether and how R1 and R2 were affected by tACS frequency, data were first subjected to a comprehensive mixed-design ANOVA with the between-subject factors *frequency* (sham, 6 Hz, 10 Hz, 25 Hz) and *stimulated hemisphere* (left, right) and the within-subject factors *session* (baseline, tACS), *target hemifield* (ipsilateral, contralateral to the stimulated hemisphere) and *contrast level* (five). Because the analyses revealed no significant difference based on the stimulated hemisphere (left, right), this factor was not further considered in the analyses.

To rule out possible differences between groups at the baseline, accuracy during the tACS session was normalised by considering accuracy during the baseline session (i.e., tACS-baseline accuracy). Accordingly, R1 and R2 normalised accuracy data were submitted to a mixed-design ANOVA testing *frequency* (sham, 10 Hz, 25 Hz, 6 Hz) as a between-subject factor and *target hemifield* (ipsilateral, contralateral to the stimulated hemisphere) and *contrast level* (five) as within-subject factors.

Although no speeded response was required, we also analysed the mean reaction times (RTs) of the correct trials of R1 for each contrast level and each frequency. Anticipations (i.e., pressing the response button before the appearance of the target), omissions (no response) and RTs shorter or longer than ±2 standard deviations of the mean of each participant were excluded from the analyses. The same mixed-design ANOVA applied to accuracy was also performed for the RTs. In all the analyses, the Greenhouse-Geisser epsilon correction factor was applied, when appropriate, to compensate for possible effects of non-sphericity in the measurements. For multiple comparisons, the Fisher's least significant difference (LSD) test was performed [57].

## Results

Most of the participants did not feel any discomfort during tACS stimulation, as revealed by their spontaneous reports and questionnaires completed at the end of the experiment. Itch and pinch were the most commonly reported sensations (in 25% and 38% of the participants, respectively) with light to moderate intensity. Importantly, there were no differences between the experienced sensations and belief about tACS's influence on the performances across participants belonging to the four stimulation groups, as shown by nonparametric Kruskal-Wallis rank sum tests (all p's>0.2).

### R1 Accuracy

**Figure 2:**
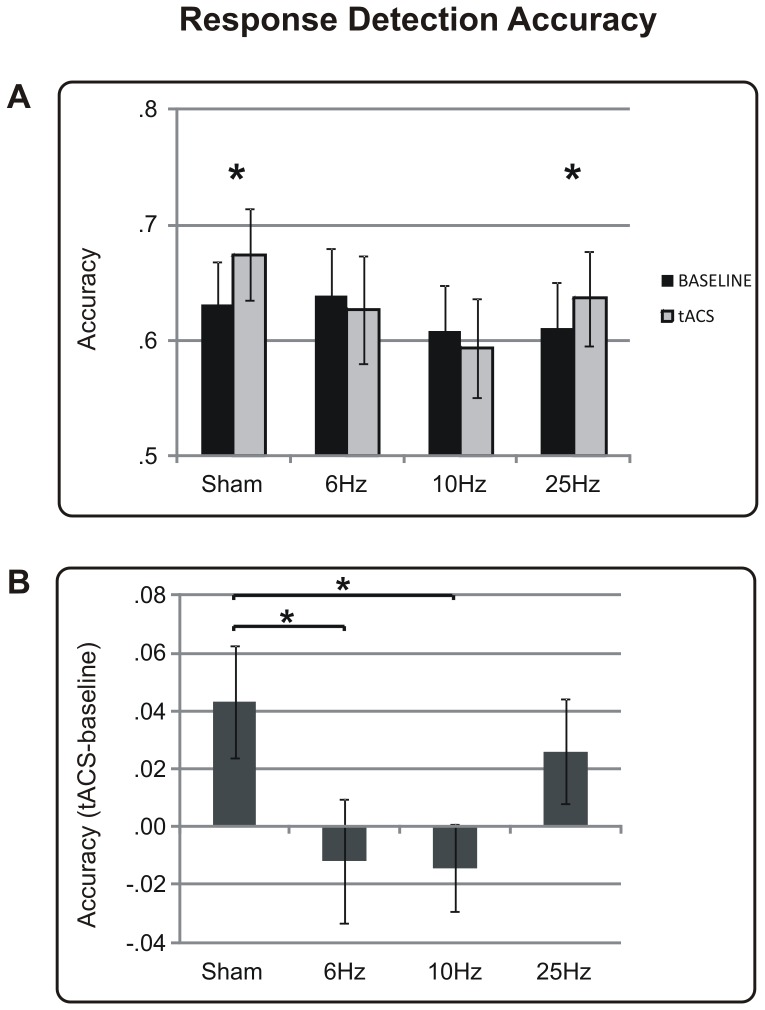
Results relative to the detection response (R1) in terms of accuracy. (A) Accuracy (proportion of correct responses) of every tACS frequency (Sham, 6 Hz, 10 Hz and 25 Hz) is shown during the baseline session (in black) and tACS session (in grey). (B) Normalised accuracy of every tACS frequency is shown as the difference between the % of correct responses during the tACS session and % of correct responses during the baseline session. Vertical bars correspond to the standard error of the mean. * indicates p<0.05.

In accordance with expectations, participants' accuracies in detecting targets showed general improvements as the contrast level increased [*contrast level* F(4,368) = 586.97, p<0.001]. R1 accuracy also improved in the tACS session in comparison to the baseline session [*session* F(1,92) = 4.56, p = 0.035], reflecting a general learning effect. This effect, however, differed between groups, as revealed by the interaction *frequency×session* [F(3,92) = 2.63, p = 0.055]. As illustrated in figure 2A, participants who received sham or 25 Hz tACS improved their performance in the second session in comparison to the first one (p = 0.017 and p = 0.024, respectively), but this improvement was not present in participants stimulated at 6 Hz and 10 Hz (p's≥0.59). This result suggests that tACS applied at both 6 Hz and 10 Hz induced a suppression of the learning due to multiple repetitions of the same task; therefore, there was no performance improvement.

A significant difference, although narrow, between groups also emerged when directly comparing the normalised accuracy of the tACS sessions [*frequency* F(3,92) = 2.63, p = 0.055]. Groups stimulated at both 6 Hz and 10 Hz showed worse performances compared to the sham group (p = 0.03 and p = 0.046, respectively) and the same trend in comparison to the 25 Hz group (p = 0.06 and p = 0.09, respectively), as shown in figure 2B.

Notably, differences between groups were always unaffected by target position because the factor *frequency* never interacted with the *target hemifield*, nor was there a significant effect due to the main factor *target hemifield* (all p's>0.1). This result indicates that the tACS-induced suppression was not specific to the contralateral side, but the suppression was generalised to both visual fields. The factor *frequency* never interacted with the factor *contrast level* (all p's>0.2), suggesting that tACS-induced effects were unaffected by the contrast values.

We also performed a mixed-design ANOVA with the between-subject factors *frequency* (sham, 6 Hz, 10 Hz, 25 Hz) and *stimulated hemisphere* (left, right) and the within-subject factors *session* (baseline, tACS) on false alarm rate to catch trials, in order to exclude changes in criterion. No significant effects were highlighted (all p>0.17).

### R2 Accuracy

**Figure 3:**
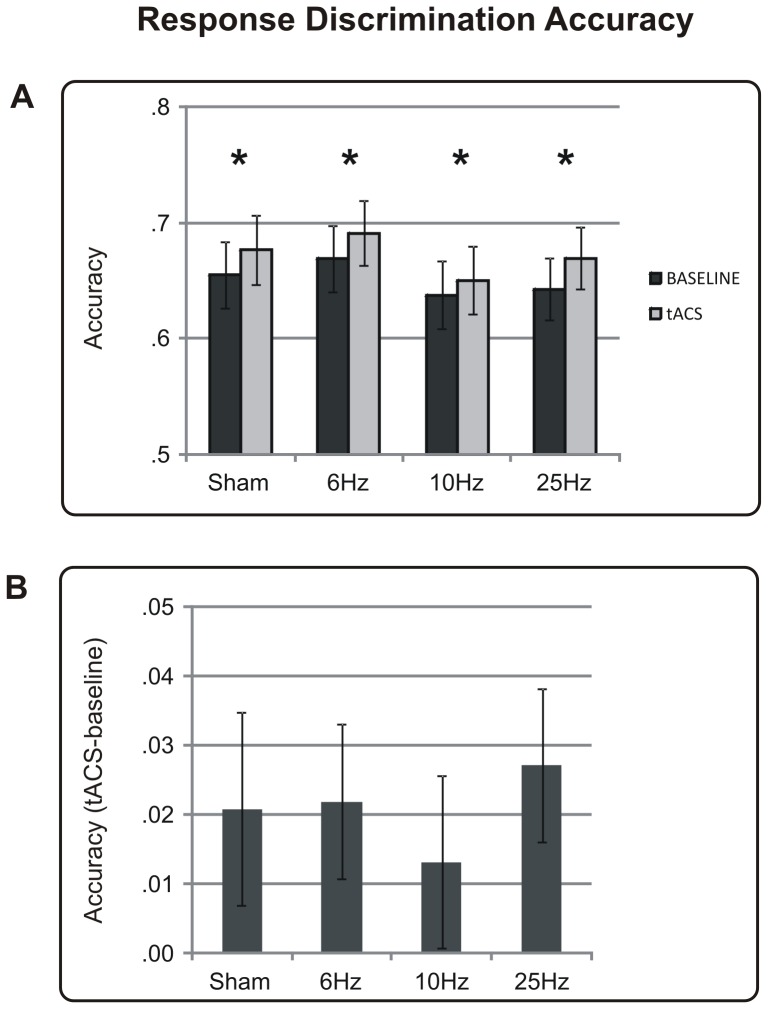
Results relative to the discrimination response (R2) in terms of accuracy. (A) Accuracy (proportion of correct responses) of every tACS frequency (Sham, 6 Hz, 10 Hz and 25 Hz) is shown during the baseline session (in black) and tACS session (in grey). (B) Normalised accuracy of every tACS frequency is shown as the difference between the % of correct responses during the tACS session and % of correct responses during the baseline session. Vertical bars correspond to the standard error of the mean. * indicates p<0.05.

When participants were asked to discriminate the orientation of the target (i.e., R2), their performances were not affected by the tACS frequency (see figure 3). Indeed, the main factor *frequency* never reached significance in the comparison between single sessions or in the analysis of normalised accuracy (all p's>0.27). As expected, R2 accuracy also improved as the contrast level increased [*contrast level* F(4,368) = 379.77, p<0.001], and the performance of all groups showed a significant enhancement in the tACS sessions with respect to the baseline sessions [*session* F(1,92) = 14.733, p<0.001], particularly at the three highest contrast levels [*session×contrast level* F(4,368) = 3.16, p = 0.02].

### Reaction Times

Because accuracy was emphasised over speeded responses, reaction times were expected to be uninformative, which was confirmed by the ANOVAs not revealing any significant effect of the factor frequency (all p's>0.3).

## Discussion

The aim of the present study was to test the ability of tACS to induce a direct oscillatory entrainment in the stimulated target area. Based on the inhibitory effects of the alpha rhythm [15], [38], [39], [40], we expected a suppression of target perception in the visual field opposite of the alpha-stimulated hemisphere. In our experiment, participants stimulated at 10 Hz showed worse performances in comparison to subjects receiving no stimulation (sham group) or high frequency stimulation (25 Hz group), partially confirming expectations. However, these effects were not retinotopically specific or frequency-specific, essential conditions that support a local alpha entrainment. Indeed, a general decrease of visual perception was observed over both visual fields, independently of the stimulation site (ipsilateral vs. contralateral), and this result was found in the groups stimulated at both 6 Hz and 10 Hz.

The spreading of the effect across hemispheres could be ascribed to the poor spatial resolution of tES, and methodological accounts have to be considered. When using rectangular-pad electrode configurations, focality is considered to be limited, and the induced effects might also be able to modulate cortical areas adjacent to the target site [50], [58], [59], [60]. Even if we properly used an electrode with reduced size for the stimulation of the occipito-parietal areas and a larger reference electrode to improve spatial focality, as suggested by Nitsche and colleagues [50] (but see [61]), we could not eliminate this possibility. The use of a cephalic reference electrode is another factor implicated in spatial focality because the relative position of both electrodes is determinant for the induced current profile in the brain [62]. However, we placed the reference electrode over the vertex, as is conventional in most previous studies on visual perception [17], [51]. In addition, the stimulation intensity adopted in this study (1 mA) could have been too low to reach the cortical target and induce a clear focal effect. However, we had to consider the possibility of inducing phosphenes by increasing the stimulation intensity, as reported by previous studies [17], [20]. The occurrence of tACS effects over both visual fields could also be ascribed to the duration of the stimulation: in each block participants received tACS for five consecutive minutes. During this period of time, current could have first directly reached the target area and then spread to the contralateral hemisphere through cortico-thalamic and/or cortico-cortical connections. Evidence for a similar mechanism has already been reported in the motor domain, where electrical stimulation was able to directly alter the excitability of the stimulated region, and indirectly, the excitability of the homologous region of the opposite hemisphere [63]. However, in this case, we would have expected the opposite effect over the homologous areas, enhancement performance in the visual field ipsilateral to the stimulation site, which is consistent with the push–pull effect previously reported regarding the posterior alpha rhythm [15], [64].

Frequency-specificity was only marginally confirmed. In the present study, precise hypotheses were formulated according to knowledge relative to the active inhibitory role of the posterior alpha rhythm during on-going visual processing [38], [39], [40]. Theta-frequency stimulation was added to the experimental design as an additional control condition, and the stimulation was expected to be ineffective [15]. Instead, participants receiving tACS in the theta frequency showed performances comparable to those of the alpha group. Although we did not intend to investigate memory and learning, the sequential design of the study (with fixed order of the baseline and tACS sessions) might explain the involvement of the theta frequency. Theta band activity, indeed, has been closely associated with memory and learning [22], [65], [66], [67], [68], [69], as well as synaptic plasticity [70], [71]. There is also evidence linking theta oscillations to other cognitive functions, such as attention [72], [73], [74] and sensorimotor integration [75], [76], [77]. Because theta band oscillations reflect long-range communication between distant brain areas [5], these oscillations have been suggested to coordinate sensory and motor brain regions when the task requires updating a motor plan on the basis of incoming sensory information. To this regard, the task performed in the present study could involve changes in theta band activity, as participants were required to have two subsequent responses according to the features of the target stimulus. Functional involvement of the theta-band activity is therefore plausible considering both learning effects and the alternating responses. Most of the previous studies, however, found a positive correlation between theta activity and task performance: increase in theta band power was observed during the encoding and retrieval of successfully remembered items [65], [78] and when the memory load was systematically increased [66], [79]. Thus, after a direct entrainment of theta by tACS, an improvement of the visual performance should be expected, instead of a worsening of target detection as in our data. Based on all of these considerations, the lack of retinotopical specificity and frequency specificity suggest that the present results may not be ascribed to the direct entrainment of brain oscillations induced by tACS. However, these data cannot prove that tACS is unable to manipulate EEG oscillations, and this uncertainty represents a study limitation.

Another point that must be discussed is that all the tACS effects observed in the present study were related to the detection, but not to the discrimination response. The difficulty level was different between the two tasks: detecting the luminance change induced by the brief appearance of the target was easier than judging its orientation. Therefore, because performance was at chance level at the lowest contrast levels in the sham condition, the second response might be less sensitive to slightly worse performance induced by tACS. However, no tACS effect on the discrimination response emerged when we focused the analysis on the three highest contrast levels, in which performance was greater than the chance level. We also analysed the discrimination responses considering only those trials in which participants had correctly detected the Gabor patch for R1 Accuracy, but no tACS effect was observed. One could argue that tACS, as applied here according to the chosen montage, actually stimulated the dorsal visual stream, where the source of alpha activity was localised [12], [80]. The dorsal visual stream is the projection conveying the signal by the magnocellular system [81], which is particularly sensitive to stimuli at low-contrast and low-spatial frequency, like those used in the present study [82], [83], [84]. Electrical stimulation of the dorsal stream could have impaired target detection because the dorsal stream responds well to rapid changes in luminance contrast. On the contrary, the fine discrimination of visual features, such as the target orientation, is also under the control of the ventral visual stream, which was not affected by the stimulation leaving the discrimination task unchanged. Nevertheless, extensive literature shows that the attention bias associated with the parietal alpha modulation affects both detection [15], [41], [46] and discrimination [34], [45] tasks. Accordingly, an effective alpha entrainment over parietal regions should have induced the same effect on both responses.

A key point worth considering in the discussion of these results is the stimulation timing relative to the phase of the on-going oscillatory activity. Brain oscillations are not only characterised by power and frequency but also by their instantaneous phase. There is compelling evidence that phase dynamics reflect cyclic fluctuations of neural excitability and play a relevant functional role in cognitive processes [3], [85], [86], [87], [88]. Schyns and colleagues [89] have recently demonstrated that phase codes considerably more information than power during an emotion categorisation task. Moreover, an increasing number of studies show that processing of visual information is strongly dependent on the phase of the spontaneous EEG oscillations, such that a stimulus appearing at the optimal phase would be optimally registered and perceived, while at the opposite phase, the stimulus might be entirely missed [90], [91], [92], [93]. Thus, studies aiming to modulate participants' behaviours through the induction of an exogenous entrainment of brain rhythms should also take into consideration the temporal dynamics of phase of the underling brain oscillations and accordingly trigger tACS application. A similar approach has been recently followed by Neuling and colleagues [94], who applied oscillatory transcranial direct current stimulation at 10 Hz while subjects were performing an auditory detection task. Importantly, they presented the stimuli in specific phase bins relative to the electrical stimulation and found specific behavioural consequences dependent on the phase of the entrained oscillation. In the present study, tACS was simply applied for five consecutive minutes without a finer synchronisation with visual stimuli, and this application could have minimised the results. Another important aspect to take into account is the individual peaks of oscillations in a particular frequency-band. We have observed that alpha frequency shows a great inter-individual variability, and the frequency also changes across life-span [95]. In our study, we did not individually select the stimulation frequency; this point is a limit of the study.

On the whole, combining all aspects discussed above with a consistent interpretation of the ability of tACS to induce a direct entrainment of cortical oscillations is quite difficult because our data did not show conclusive proof or disclaimer of the point. Perhaps the most parsimonious explanation could be found by considering the possibility that the current flow spreads through the retina. Even if tACS did not induce a conscious perception of visual phosphenes in the present study, its action, which is subthreshold by definition due to the low stimulation intensity, could still affect the functioning of retinal cells. In this regard, we consider that contrast detection starts from the retina while orientation discrimination is a cortical process that occurs at the level of the primary visual cortex (i.e., V1). Indeed, research has established that different aspects of a visual scene are processed by separate parallel pathways, which run from the ganglion cells of the retina to the V1, passing through the lateral geniculate nucleus [96], [97]. The magnocellular and parvocellular cell systems differ significantly in their anatomical and physiological properties [84]. In particular, they differ with respect to contrast gain: the former systems are much more sensitive (8–10 times) to luminance contrast than the latter [82]. Thus, the magnocellular system is well suited to handle detection of rapid changes of low luminance stimuli, and this system is already in the retina. The recognition of stimuli orientation, instead, occurs in the V1 cortex, where the information is carried by both the magnocellular and parvocellular systems [96]. The results of the present experiment could be explained if tACS at low frequencies (6 Hz and 10 Hz) was able to selectively interfere with the magnocellular but not the parvocellular cells of the retina. In this case, detection would be impaired, while discrimination could be supported at the V1 level by the parvocellular system. Studies on the primate retinal ganglion cells showed that cells in the magnocellular system actually have temporal-frequency response characteristics distinct from cells in the parvocellular system [98], [99] and they peak at approximately 10 Hz [100]. Although intriguing, this theory is only a speculative explanation that needs to be investigated in further studies.

Another concern with tES in general (not only tACS) regards the way in which current flows through the brain. While the effects induced by electrical stimulation directly applied to the cortical tissue are well established [101], [102], [103], [104], [105], [106], [107], the same is not true when stimulation is applied transcranially over the scalp. During any tES modality, the current that reaches the cortex is strongly influenced by anatomical factors because of the different electrical conductivities of the intermediate tissues, such as the scalp, skull, cerebrospinal fluid and brain [59], [108]. Moreover, because the impedance of the skull is higher relative to that of the scalp, most of the current is shunted across the scalp [60]. Evidence provided by imaging and modelling studies [30], [31], [59], [109] as well as clinical studies [110], [111], [112] suggests a widespread modulation of multiple cortical and sub-cortical regions, independently of their anatomical connections. Considering these aspects, the range of possible interpretations for the data of the present study becomes wider and more elaborate.

In conclusion, the present study does not provide decisive evidence for tACS reliably inducing direct modulations of the natural brain oscillations in a visual detection and discrimination task. Although previous results appear to support this possibility [18], [21], [22], [26], data from this study lacks the retinotopical-specificity and frequency-specificity necessary to conclusively argue for the capability of tACS to modulate spontaneous brain oscillations. On the whole, we urge caution and the need for further investigation.
